# Measuring health promotion: translating science into policy

**DOI:** 10.1007/s00394-020-02359-1

**Published:** 2020-08-27

**Authors:** James C. Griffiths, Jan De Vries, Michael I. McBurney, Suzan Wopereis, Samet Serttas, Daniel S. Marsman

**Affiliations:** 1 Council for Responsible Nutrition-International, Washington, DC, USA; 2 Nutrition in Transition Foundation, Gorssel, The Netherlands; 3 Department of Human Health & Nutritional Sciences, University of Guelph, Guelph, ON N1G 2W1 Canada; 4 Research Group Microbiology and Systems Biology, Netherlands Organization for Applied Scientific Research (TNO), Utrechtseweg 48, NL-3704 HE Zeist, The Netherlands; 5 Herbalife Nutrition, Ankara, Turkey; 6 Procter & Gamble Health Care, Cincinnati, OH USA

**Keywords:** Ageing, Health promotion, Healthspan, Microbiome, Lifespan, Nutrition

## Abstract

Commonly, it is the end of life when our health is deteriorating, that many will make drastic lifestyle changes to improve their quality of life. However, it is increasingly recognized that bringing good health-promoting behaviors into practice as early in life as possible has the most significant impact across the maximal healthspan. The WHO has brought clarity to health promotion over the last fifteen years, always centering on language relating to a process of enabling people to increase control over, and to improve, their physical, mental and social health. A good healthspan is not just freedom from morbidity and mortality, it is that *joie de vivre* (“joy of living”) that should accompany every day of our lifespan. Therefore, health promotion includes not only the health sector, but also needs individual commitment to achieve that target of a healthspan aligned with the lifespan. This paper explores health promotion and health literacy, and how to design appropriate nutritional studies to characterize contributors to a positive health outcome, the role the human microbiome plays in promoting health and addressing and alleviating morbidity and diseases, and finally how to characterize phenotypic flexibility and a physiologic resilience that we must maintain as our structural and functional systems are bombarded with the insults and perturbations of life.

## Introduction

Over the last four years, the Council for Responsible Nutrition-International (CRN-I) has endeavored to significantly add to the body of science through their focus on orchestrating and moderating a series of expert presentations, with concomitant publications, held at the annual Codex Alimentarius (Codex) Committee on Nutrition and Foods for Special Dietary Uses (CCNFSDU). The most recent topics are inter-related and have covered optimal nutrition [54], healthy ageing [28, 37], and in this most recent iteration, concepts around health promotion. These previous publications included perspectives from the World Health Organization (WHO), and the symposium which spawned this conference report was also presented against a backdrop of the WHO’s activities from the Department on Health Promotion, summarized here from publicly available WHO materials.

## Health promotion

The WHO’s Department on Health Promotion envisions “a world which is free of health inequalities and preventable disease caused by risks and other determinants of health and where all people enjoy well-being through health protection and promotion, preventive actions and healthy life choices” [67, 72]. To accomplish this, WHO seeks to “contribute to people enjoying better health and wellbeing, and reduced health inequalities, through a coordinated inter-sectoral approach acting on health determinants” [69]. But there is not a magic wand nor an instantaneous “cure”. The Ottawa Charter of 1986 stated that “health promotion is the PROCESS of enabling people to exert control over THEIR health and their determinants” [emphasis added; [66, 71], i.e., enable each consumer to have control over the choices that will determine the trajectory of their health to the benefit (or detriment) of the individual and society as a whole. In the quarter century that has elapsed, that Ottawa statement still rings true. Of late, there is movement away from only using the disease state, or conversely, freedom from disease, as the arbiter of what constitutes health and healthspan, and that one needs to focus on the ‘valued outcomes’ that arise from health promotion [43, 55]. The Shanghai Declaration of 2016 further highlighted the role of health promotion in the WHO 2030 Agenda for Sustainable Development [70] as a necessary step to achieve healthy ageing, suggesting that healthspan and not just chronological age should be aligned with lifespan [74].

When health promotion has been encouraged and becomes ingrained into the population, then the desirable lifestyle outcomes are realized, leading to decreased morbidity and disability, functional independence and the desired quality of life that define a good healthspan through a person’s lifespan.

## Health literacy

There are several levels of increasing literacy when it comes to health promotion, and it is important to characterize these so that messaging can be developed to cover all levels of understanding, and to help move a person’s ability to comprehend from the most basic to ever more complex layers of information that will in the long run help make health-promoting choices more likely.

### Functional health literacy:

Refers to the basic skills in reading and writing, as well as basic knowledge of health conditions and health systems needed to obtain health information and comply with this information.

### Communicative health literacy:

More advanced literacy and personal skills required to access, understand and discriminate among health information from different sources, and independently apply new information to changing circumstances.

### Critical health literacy:

The most advanced cognitive and social skills which enable people to critically analyze health information from a variety of sources and use this information to exert greater control over personal health decisions and the wider influences on those decisions [44].

To apply these levels of literacy to a real world nutritional situation, one can think of functional literacy as being able to read a nutrition or supplement facts label to determine the number of calories or amount of vitamin D in the product. Communicative literacy would allow one to compare products and to prioritize which would be the healthier option based on a single nutrient, e.g., fewer calories or higher levels of vitamin D. Critical literacy would integrate information from many sources, recognize trade-offs, and be able to prioritize the most desired option for the individual which may not be applicable to everyone, perhaps higher calories but also higher vitamin D, as the latter is of more concern to that individual, especially as one comprehends the role of the totality of one’s diet in meeting nutrient needs.

## Non-communicable diseases

WHO and Codex and many nations use the term “Non-Communicable Diseases (NCDs)” to describe what the general population might call “chronic diseases”, i.e., those long duration debilitations that result from a combination of genetic, physiological, environmental and behavioral factors. The most common causes of morbidity and mortality are NCD’s, including the spectrum of cardiovascular diseases (stroke and heart attacks), cancers of all types, diabetes, and chronic respiratory diseases (asthma and chronic obstructive pulmonary disease).

Historically, NCDs disproportionately affected people in low- and middle-income countries that had poor dietary options, but of late, it is now known that some NCDs are a great equalizer, as individuals living in developed countries with a lifestyle characterized by nutrient-poor diets, high rates of smoking and alcohol use, lack of physical activity and rising psychological stress are being affected with NCDs, such as diabetes and obesity, at an alarming rate. In the words of a colleague, NCDs are “the most democratic of diseases, affecting all populations in all countries.”

According to WHO, NCDs kill approximately 41 million people each year, equivalent to 71% of all global deaths. Cardiovascular diseases account for most NCD deaths, with 17.9 million people annually, followed by cancers (9.0 million), respiratory diseases (3.9 million), and diabetes (1.6 million) with numbers still on the rise [73].

**Fig. 1:**
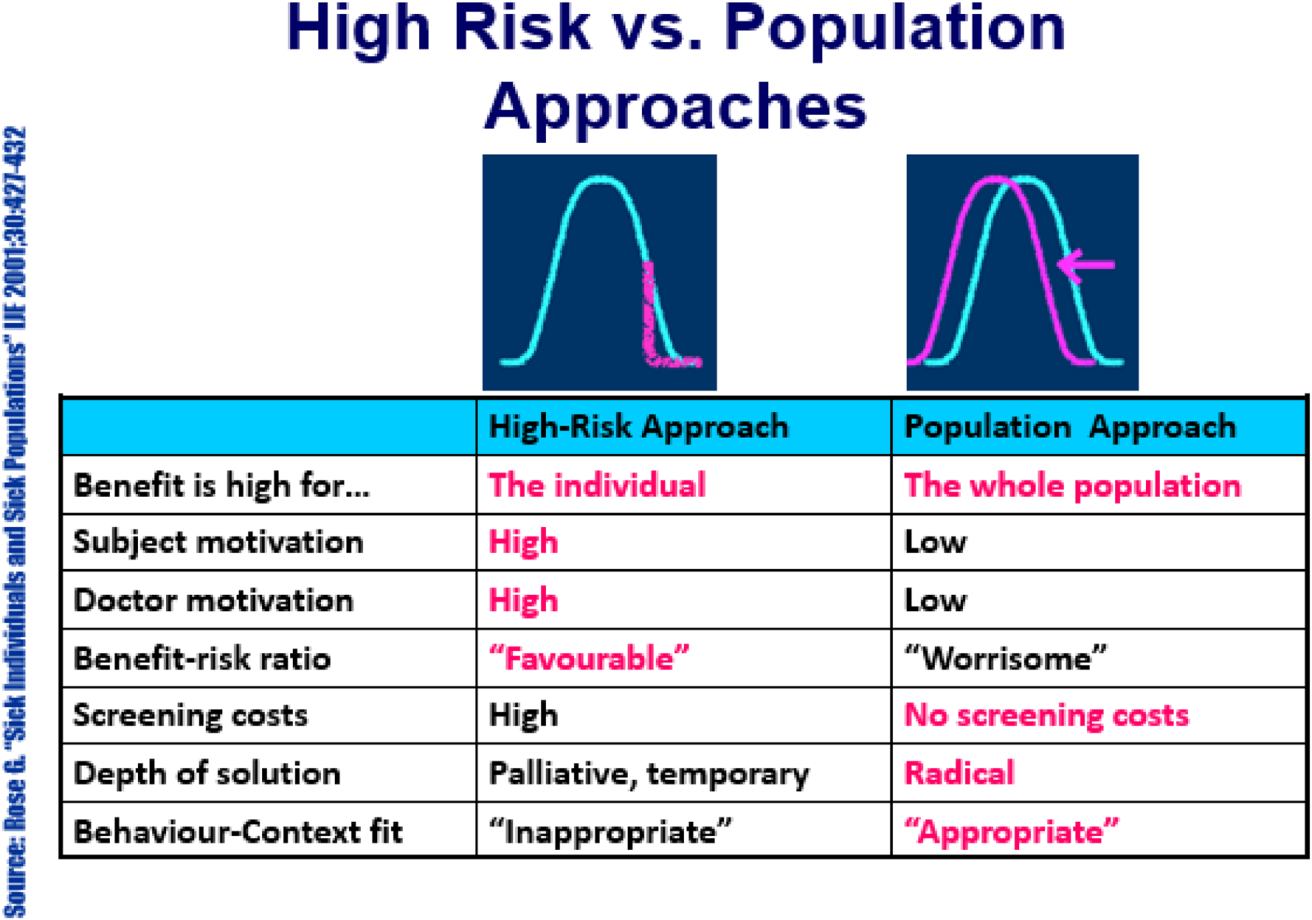
Individual high-risk approach versus a population approach [50] (Rose, Sick individuals and sick populations, Int J Epidemiol, 2001, 30(3), 427–432, by permission of Oxford University Press)

It is clear that a global action plan is needed to shift the paradigm to prevention and control of NCDs, but health literacy is insufficient. Where does one invest in the principles of health promotion—at the individual or societal levels (Fig. 1) [50]? If one targets the high-risk individual, then there is a benefit to just that person, assuming a high level of motivation and compliance, but the expense is high and the solution may only be temporary, as the common environmental risk factors (e.g., nutrient-poor diets) remain. At the population level, the majority benefits, but the individual motivation may be low, but the solution—if achieved—is radical. Regulations that lower salt and sugar content could benefit society, but there are outliers including “damn-the-torpedoes-individuals” who continue to dismiss the risks of a dangerous action.

WHO convened a meeting in 2015 to consider the scope, design and implementation of effective fiscal policies on diet and health promotion [68]. It was concluded that there is reasonable and increasing evidence that appropriately designed taxes that raise the prices of sugar-sweetened beverages (SSB) by > 20% could lead to more than a proportional reduction in SSB consumption and net reductions in caloric intake. Further, subsidies for fresh fruits and vegetables that reduce their prices by 10 to 30% are effective in increasing fruit and vegetable consumption. By a combination of both effective policies, implementation of sugar taxes along with nutrient-dense food subsidies, the health-promoting effect is even greater. However, as mentioned, societal approaches, such as taxes or subsidies, carry a substantial societal cost, and regulations mandating changes to products with perceived better nutrition generally lack sufficient long-term incentive for an individual to comply. As another example, smoking is still a health scourge, even with ever-increasing taxes and age restrictions that result in consuming more of the personal budget, perhaps limiting money for better dietary choices, i.e., an exacerbated situation.

This paper will delve into new concepts and technologies in measuring health promotion by examining how nutrition science has and may play a role in better understanding and/or communicating the baseline and measures of an intervention effect to determine if there is movement in the right direction. Nutrition science is more than just a set of objective measures; there are the subjective aspects of behavior and culture that play important roles in communicating health promotion. Nutrition science is complex and multi-disciplinary making academic pursuits complicated. New concepts to quantify health effects of food and nutrition, such as “resilience” and the gut microbiome, are being established and leading to the development of a next generation of biomarkers that may be used to substantiate beneficial health effects. New self-monitoring technologies may lead to a more personalized public health and nutrition modality, allowing for population approaches with a personal incentive for individual compliance.

## Credibility and capability of nutrition sciences in the perspectives of health promotion

In the past decades, scientific efforts elucidating the relation between nutrition and health have greatly increased our understanding, for example, *trans*-fatty acid intake and coronary heart disease. By direct extension, these efforts have raised societal awareness about the connection between diet and health. However, recently the general trust in nutrition sciences appears to be declining. A Dutch collective of nutritionists and medical doctors, suggested that nutrition science is meeting inherent boundaries, hampering conceptual and methodological progress and the translation of novel insights into societal benefit and trust [47]. Others have initiated activities to gain insight on how experts in nutrition science can improve their credibility and capability [22, 29]. In other words, many scientists consider nutrition science is facing its limitations with capability and credibility, impeding its societal value. This is very unfortunate, as we now face the global challenge of developing sustainable and healthy food systems allowing future food security that supports healthier life opportunities for all.

The approach to studying the effects of diet on health needs to change. Much of the challenge involves the redefinition of concepts of nutrition science, such as the definition of health and how to determine health status (of the individual, societal groups, nations, the planet), what to consider to be a causal effect and the process of achieving consensus based on the totality of the evidence on causality. Recently, a more detailed concept of health for the nutrition sciences was championed during a satellite workshop to the FENS conference [7, 17, 59]. At the same workshop, it was acknowledged that the quality of nutrition science should be at the highest level, however, taking into account that one must accept the fluidity of knowledge and that the spectrum of certainty will never be binary, either “A” or “B”.

The reductionist approach, which does have a binary character, has proven to be indispensable to answering questions related to specific ingredients and improving mechanistic biochemical understanding of the effects of single components, most notably vitamins. This approach led to the discovery of vitamins and their role in human health, or what their absence means to lack of human health. However, emphasis on thinking in terms of individual substances and explaining effects only by one-on-one molecular mechanisms have become so ingrained as to hamper nutrition science’s ability to diversify its views on public health nutrition guidance beyond the statistical or biochemical behavior of single molecules. The often a priori exclusion of nutritional factors in studies on the treatment of disease further undermines the important impact that nutrition plays in promoting an optimized healthspan.

The randomized controlled trial (RCT) has become the highest ranked tool in the evidence pyramid [51]. However, the double-blinded 2 × 2, short-term design cannot be applied to foods, meals or dietary patterns. Results from RCT’s, therefore, need to be translated to make hypotheses regarding the complexity of foods and daily diets in the context of daily behavior. This translation has serious pitfalls because it often extrapolates short-term intervention studies to lifelong expectations and outcomes. What is a proper placebo in relation to the intervention (and is it ethical to have a RCT arm lacking in a putative beneficial moiety?)? Did the study involve biomarkers? Were these biomarkers validated or scientifically accepted? Are these biomarkers effective for long-term health assessment? Nutrition RCT’s may represent a high level of internal validity but the external validity often fails when it comes to adequate nutrition.

Methods in nutrition science need to change to accommodate the questions related to the challenges resulting from the differences between internally to externally valid research results. The real-life nutrition conditions are not dependent on single ingredients or products, but more on a proper understanding of the concept of a “balanced diet”, which may have many faces, as human metabolism demonstrates a large capacity for flexibility. For example, is the health outcome a response to changes in energy balance, the relative proportion of energy components (fat, carbohydrate, protein and alcohol), or the abundance of essential or semi-essential nutrients (amino acids, long-chain fatty acids, vitamins, minerals)? The *relevance* and *impact* of nutrition science primarily consist in the increased knowledge about the long-term impact of nutrients, foods and food patterns on health maintenance and disease onset. This needs an expansion into adjacent scientific fields beyond the biomedical domains, such as the social sciences and data sciences, to better understand what drives human beings to the foods they want. Meanwhile, we are facing a global transition in food production: how to feed the expected 10 billion individuals in 2050? This needs to be done in a sustainable and affordable way and should be taken into account in future nutritional science.

What changes are needed to create capable and credible nutrition science? It is clear that nutrition sciences are not only about the biochemicals. It includes cultural, behavioral, environmental and sustainability elements. The scientific challenges are at transdisciplinary, multi-level and intergenerational level requiring multi-sectoral and multi-stakeholder inputs in an area with a shifting paradigm. A better understanding of the concept of health with the metabolic flexibility that humans demonstrate is highly required. Improving this understanding will need the use of new technologies, often referred to as Big Data, as well as high and consistent standards in data quality. Analyzing these data requires new and strong data sciences, statistics, and maybe even artificial intelligence that allows the integration of an almost infinite number of data points. Furthermore, the combination of social sciences and life sciences needs to adopt qualitative and quantitative methods that are thoroughly validated allowing the development of transdisciplinary and open sciences (FAIR (Findable, Accessible, Interoperable, and Reusable) data disciplines [75].

**Fig. 2:**
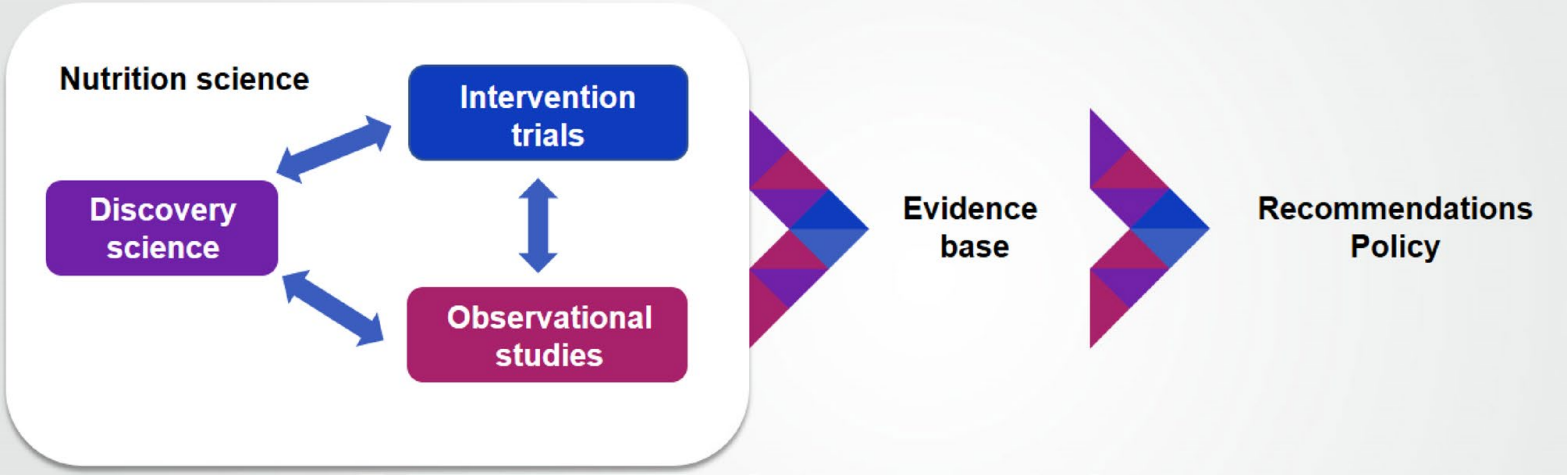
Circle containing discovery sciences, observational studies and intervention studies

To understand the factors affecting human behavior and health and societal outcomes, it will require basic research (discovery science), observational and intervention studies. From this circle [Fig. 2] of scientific activities, evidence-based information is required to set credible recommendations to the public on how to eat and apply lifestyle choices to enjoy healthy lives. As scientists, we know that facts are the foundation of science but the general public and policy-makers often think things are only true or not true. Public trust will increase when nutrition science can credibly translate facts into public health recommendations that are well understood and accepted within society. Only then will literate and actionable information be conveyed to the individual, the group, the society and the planet.

The general public is interested in food matters, usually three times every day. Commercial nutrition, confusing health claims and scientific conflicts of interest unfortunately lead to mixed and contradictory messaging, the result is a genuine and hard-to-resolve level of distrust of scientists by the public. The moral character and reliability of nutrition science and its champions seems to be at stake. The Dutch Nutrition in Transition (NiT) initiative is evaluating the effects of what we eat on individual and public health [45]. Recently the Federation of European Nutrition Societies launched several working groups under the umbrella of “Improving Standards in the Science of Nutrition”. The first working group will focus on concepts in nutrition science needing revision and how this can be achieved, to have credible and capable scientific research. A second working group will focus on the organizations, the capabilities and the funding for nutrition sciences. A third working group will assess what is needed to translate scientific findings into believable and proficient recommendations to regain public trust. The working groups intend to finalize and report on their activities at the Federation of European Nutrition Societies’ (FENS) meeting in 2023.

## Defining a healthy microbiome: lessons learned from nutrition

The human gut microbiome is composed of the totality of the genetic material of all microbes—bacteria, fungi, protozoa and viruses—that live in a person’s gastrointestinal tract. The assembly of the gut microbiome is associated with immune system development, risk of infections, nutrient acquisition, and potentially brain and nervous system functionality. Much is currently being said in science and communicated by the media about the role of the gut microbiome in human health. The next section will review definitions and factors affecting the human gut microbiome from birth onwards. Finally, applying examples from nutrition, we will discuss scientific questions needing answers to define a healthy microbiome.

The human gut begins to be colonized by microbes during birth. Intestinal colonization proceeds in an incremental manner and is affected by mode of delivery (vaginal versus Cesarean-section), diet (human breast milk, infant formula, introduction of solid foods), probiotic supplementation, and antibiotic use. Gut microbiome assembly is associated with immune system development, risk of infections, nutrient acquisition, and potentially brain and nervous system functionality [20]. Human diseases are often associated with a ‘dysbiosis’ of the gut microbiome, i.e., an altered composition or functionality in subjects with diseases compared with healthy controls. Causality has not yet been established, so technical challenges in establishing a healthy human gut microbiome, including lessons learned from the application of validated biomarkers of structure/function outcomes for vitamins and dietary fiber claims, will be reviewed with the goal of identifying the type of information needed to define a healthy gut microbiome and to establish healthy gut microbiome–host relationships, factors that lead towards a health-promoting gastrointestinal community.

The human gut begins to be colonized during birth and develops into a mature, stable equilibrium with a diversity of organisms interacting synergistically with the host to help maintain health [41, 48]. The composition and structure of the adult gut microbiome is subject to microbiome-intrinsic factors (age, disease, compositional state, stochastic and founder effects that affect taxa interactions); environmental factors (local environment, regional strain pools, household and family factors that affect vertical transmission); host-extrinsic factors (diet, medication, cultural habits, physical activity, intestinal transit time); and host genetics (adaptive and innate immunity, sex, body mass index) [52]. Disruptions to the structure and function of the microbial community can occur, i.e., dysbiosis, and researchers are trying to understand if these changes proceed disease, are a consequence of disease, or if both occurrences are possible [48].

Delivery mode affects the development of the infant microbiome. Differences are observed in the structure of the initial microbiome between children who were born vaginally versus by Cesarean [15]. As children grow, the gut microbial community structure differs between those receiving breast milk or formula at 3 months and with the subsequent introduction of complementary food [19]. Antibiotic use in healthy adults for 4 days reduces gut microbiota richness and recovery is still incomplete 180 days later [46]. Strain-level differences in gut microbiome diversity are found among omnivores, vegetarians and vegans with an increased prevalence of *Prevotella* strains with enhanced potential for carbohydrate catabolism when diets are rich in fruit and vegetables [11]. Epidemiological immigration studies report a loss of microbiome diversity, a displacement of non-digestible carbohydrate (dietary fiber) digesting organisms (*Prevotella* strains) by *Bacteroides* strains, and a loss of bacterial enzymes for dietary fiber digestion in the residual *Prevotella* strains when people move from Thailand to North America [64]. These changes may be the result of a diet lacking plant-based cell walls, i.e., dietary fiber, to maintain a healthy gut microbiome [35].

### Strategies to support a healthy gut microbiome

1. Rebalance with beneficial probiotics
  A probiotic is a live organism which when administered in adequate amounts confers a health benefit on the host [24]. For a probiotic organism to successfully establish within the gut, it must: (1) be introduced (ingested or fecal transplant), (2) establish as a viable population, (3) grow and persist upon the available resources, and (4) affect microbiome composition and function through competition, antagonism or mutualism [65]. There is direct evidence that orally-administered probiotics can persist in the gut for 6 months [36]; however, engraftment does not necessarily alter the resident microbiota composition and it may be dependent upon endogenous organisms and their capacity to compete for resources.
2. Prebiotics: Feeding the Beneficial Microbiota
  A dietary prebiotic is a substrate that is selectively utilized by microorganisms such that the gut microbiome is modulated to confer a beneficial health benefit to the host [23, 30]. As an example, galacto-oligosaccharide supplementation for 12 weeks increases *Bifidobacterium* numbers [8]. Several reviews have been published on prebiotic dietary fibers [9, 18, 77].

McBurney et al. [39] summarized the two types of dietary fiber-related health claims: product claims and efficacy claims. Product claims require measurement of an ingredient or component in a food or supplement, e.g., 3 g of prebiotic dietary fiber per 20 billion CFU per serving. Efficacy claims, e.g., help to maintain a healthy digestive system, require effective biomarkers that can be objectively measured and evaluated as an indicator of biological processes in the host. This is the approach used to define dietary requirements for iron and vitamins [3, 34, 58]. Surrogate endpoints that are predictive of disease are also used, e.g., LDL-cholesterol for cardiovascular disease, blood pressure for hypertension, and hemoglobin A1c for diabetes. Regulatory authorities also use these biomarkers and surrogate endpoints to evaluate efficacy of nutrition interventions in clinical trials [4, 26, 39].

Because of the considerable microbial taxonomic diversity in stool samples obtained from individuals living in countries around the world [12, 21], it is difficult to define ‘normal’ or ‘healthy’. Moreover, fecal microbial diversity can change (1) with season as demonstrated in studies of Hadza hunter-gatherers living in Tanzania [21] and (2) dependent upon socio-economic status/neighborhoods of Chicago [40]. Stool microbiome diversity changes daily, is related to diet history, and similar foods have different effects on different people’s microbiome [27].

Before a healthy gut microbiome can be established, scientific consensus on the characteristics of a healthy microbiome is needed. Is a healthy microbiome defined by microbiota composition (richness), diversity (balance), stability or resilience to perturbation, microbial function (metabolism, end products, etc.) or some algorithm considering each of these factors? Then, to determine a health benefit for an outcome of interest, scientific consensus on effective biomarkers or surrogate endpoints for the outcome is needed. This requires understanding the mechanisms of action (X affects Y) and scientific validation. Finally, given the diversity of the gut microbiome observed among humans globally, it is critical to understand if dysbiosis causes disease, is a consequence of disease, or if both occurrences are possible.

## Phenotypic flexibility and health promotion

**Fig. 3:**
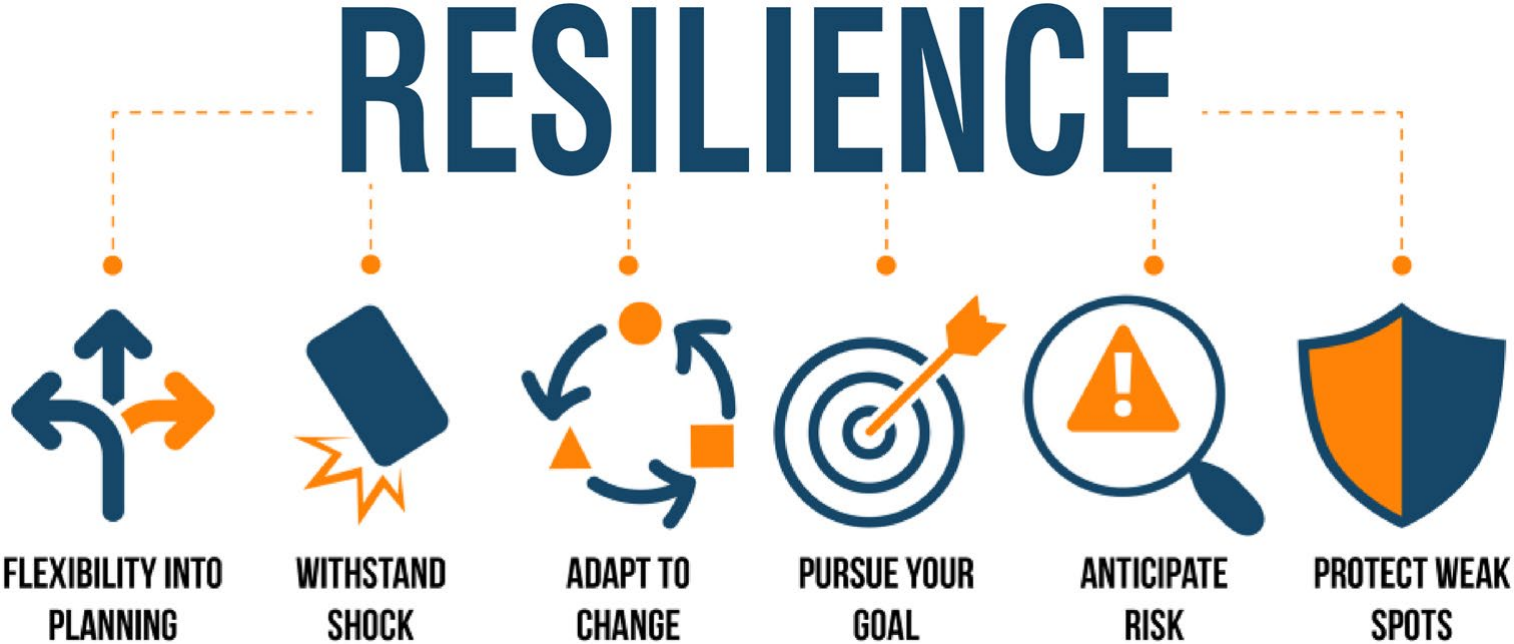
Resilience [49] (Research on Investment, www.researchoninvestment.com/resilience-in-the-face-of-change/, 2020, by permission of Research in Economic Development)

Heath promotion is also dependent upon the ability of a person to maintain or regain homeostasis. This can be termed “resilience”, per the dictionary “the power or ability to return to the original form, position, etc., after being bent, compressed, or stretched; elasticity” [13]. The following figure (Fig. 3) comes from a paper on resilience in economic development…but it is just as applicable to human resilience to being “bent, compressed or stretched” and the ability to achieve a health-promoting lifestyle. How can one measure or quantify resilience [49]?

Methodology has been developed to examine ‘phenotypic flexibility,’ that is the resilience expressed as the cumulative ability of all one’s physiological processes to return to homeostatic normality after a short-term perturbation.

In nutrition and health science, the focus is shifting from disease prevention towards health optimization and wellness. However, health and nutrition sciences have struggled to demonstrate or measure effects resulting from health promoting actions/activities.

**Fig. 4:**
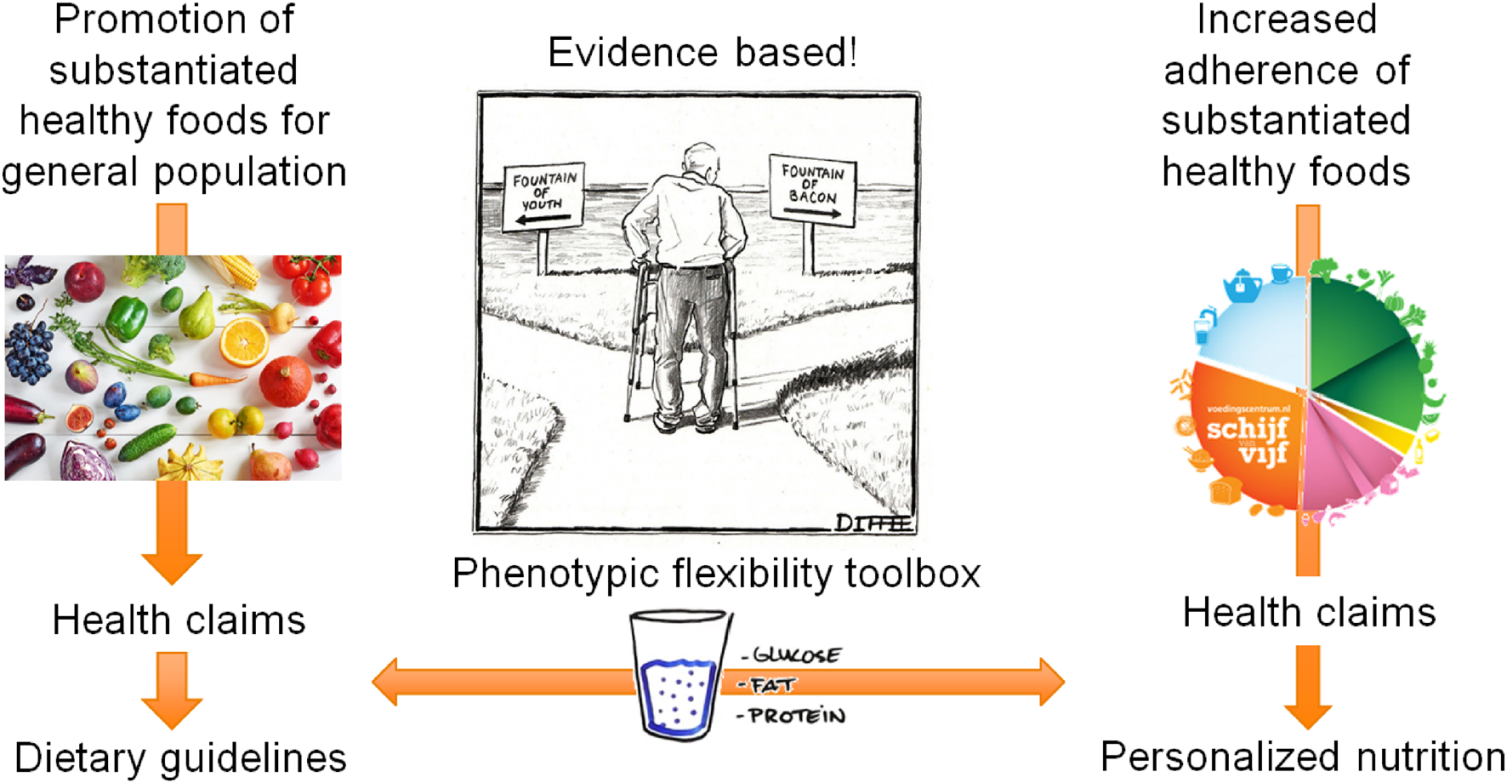
Two routes can be distinguished for evidence-based health promotion as represented by the fountain of youth: (1) Through the promotion of healthy foods for the general population by health claims. Ideally foods with a health claim (structure function, function claim and disease risk reduction claim) will also be adopted by national and international dietary guidelines; (2) Through increased adherence of the general population to healthy diets and foods by means of personalized nutrition. Through personalized nutrition, people may adhere to substantiated dietary guidelines as well as to foods with substantiated health claims. Quantification of phenotypic flexibility may allow one to substantiate health effects of food and nutrition to generate next generation health claims as well as quantify health effects from application of personalized nutrition

From a scientific perspective, two different routes for evidence-based public health promotion can be distinguished (Fig. 4). The first one is the promotion of substantiated healthier foods with a structure–function (US) or function claim (Canada and EU) for the general population [39]. This can be augmented by increasing the number of foods and food products with a disease risk reduction claim linking the consumption of the food with a reduction in risk of developing a diet-related disease or condition [39]. Ideally, the foods with a disease reduction claim will also be adapted into the dietary guidelines for public health promotion, perhaps with incentives to choose those healthier food options. The second route for health promotion from an evidence-based perspective is the increased adherence of the public to substantiated healthy dietary patterns and/or foods. This may be realized by means of a personalized nutrition approach. The quantification of health and health effects via ‘phenotypic flexibility’ may help to substantiate subtle personal health effects of nutrition and, therefore, can help in both routes for promoting health. In the following paragraphs, it will be explained what we understand by health quantification through the assessment of phenotypic flexibility as well as the explanation of how phenotypic flexibility can help with both routes for health promotion.

24 h a day, seven days a week, people cope continuously and subconsciously with changes in their environment, including the intake of foods, the levels of physical exercise, stress, etc. Their ability to adapt to perturbations can act as an indicator for maintenance or improvement of physiological function. The individual`s capacity to adapt to alterations in dietary conditions is called “phenotypic flexibility”. Phenotypic flexibility can be defined as the metabolic adaptation to a disturbance of homeostasis by a series of interconnected physiological processes and molecular mechanisms [62]. Phenotypic flexibility inherently describes health as a dynamic situation, addressing the constant efforts of physiology to maintain homeostasis of the body, thus acting like a shock absorber. In this view, nutrition has the primary role to deliver calories and macronutrients and the subsidiary role to deliver other essential nutrients, which can be regarded as the lubricants of physiology. Prolonged unbalances in nutritional intake will compromise the resilience of the physiology, reducing its ability to cope with daily stressors and ultimately creating a risk situation for disease development. In new intervention studies, phenotypic flexibility is tested by applying standardized dietary or other challenges, followed by determining the amplitude and recovery time of the responding markers. Useful markers can be (combinations of) any relevant quantifiable biological parameter [61, 63].

Since the introduction of the so-called health claims directive, which amongst others, entails functional health claims and disease risk reduction claims, in the European Union (EC1924/2006), most health-claim dossiers from the food industry have been rejected by the European Food Safety Authority (EFSA) mainly due to shortcomings in demonstrating cause–effect relationships [38]. Effects of dietary interventions are difficult to put into the context of ‘health’, since assessment of cause–effect relationships and assessments as to whether the observed effect can be convincingly considered as a true health benefit, are lacking in most cases. Ultimately, “improved resilience” (or related wording) may become a new EFSA-accepted claimable health benefit for food.

Therefore, the following route was taken to work towards next-generation biomarkers based on the concept of phenotypic flexibility that could be one day accepted by regulatory authorities, such as EFSA. Based on an extensive literature review, a standardized challenge test called the PhenFlex test (PFT) was developed which relates to the concept of the body’s “ability to adapt” as a measure for health [57]. On the basis of a collection of a multitude of biomarker-response profiles reflecting defined and accepted biological processes that are interconnected in the ‘systems flexibility network’, it was investigated as to what extent these response profiles can determine health. Therefore, a number of human volunteer studies have been performed that showed that the amplitudes of these biomarker responses to PFT clearly differentiated between individuals with optimal resilience or predisposed to a disease, as well as with metabolic disease [60, 76].

As the PFT approach has been standardized, it can now be used to scientifically demonstrate individual health effects and the effect of single food products on health. Due to reviewing the relative contribution of each of the initially broad panel of biomarkers in the previous studies, now a subset of biomarker responses can be used which are most important in measuring a certain health area or for a certain food product. Importantly, this indicates the possibility that recovery of homeostasis (resilience) can indeed be regarded as a new measure of an individual’s health.

In a first proof of concept, we were able to show a beneficial effect on liver and inflammatory resilience after a 12 week study that exchanged a diet of refined wheat for a whole grain wheat diet [25]. Besides evaluation of the effect of the intervention on single markers in response to PFT, 3 composite markers were also created based on a multivariate health space visualization model containing all markers and time points focusing on (1) metabolic resilience (based on a total of 6 markers); (2) liver resilience (based on a total of 4 markers) and (3) inflammatory resilience (based on a total of 4 markers), allowing one to evaluate the intervention effects on the ‘body function’ instead of on separate single markers. Interestingly, the intervention effect on liver resilience could be confirmed by intrahepatic liver lipid contents, which indeed showed that whole grain wheat prevented liver steatosis [53].

Meanwhile, the concept of resilience as a monitor for a health benefit has been accepted by the EFSA Scientific Committee [16]. The results of the proof of concept and the resilience composite biomarkers were discussed with EFSA health claim dossier consultants. Based on this evaluation, we are currently working on providing the scientific substantiation dossier of these markers of resilience by delivering (1) the scientific rationale that defines resilience as a specific body function, (2) why improvement of resilience should be considered a beneficial physiological effect and (3) an explanation on why the combined biomarker concept is an appropriate measure of resilience.

The second route for health promotion from an evidence-based perspective is the increased adherence of the public to substantiated healthy dietary patterns and/or healthier foods via personalized nutrition as reviewed by Adams et al. [1]. They describe personalized nutrition as the use of individual-specific information, to promote dietary behavior changes that may result in measurable health benefits. Individual-specific information includes but is not limited to socio-environmental factors, personal behaviors, phenotypic and/or genotypic parameters, that is more specific than information on the population level. Founded in evidence-based science means that the tools used for individual data collection, as well as any nutritional advice that is being provided to the individual meets the well-established rigor and reproducibility principles of scientific substantiation. A critical step in personalized nutrition is the promotion of a change in individual dietary behavior ideally resulting in quantifiable health benefits to the individual, but also to the entire population subjected to personalized nutrition.

The field of personalized nutrition is relatively young. The first studies show that the application of personalized nutrition indeed results in changes in dietary habits resulting in quantifiable health benefits to a higher extent as compared to generic advice. The most well-known study in this area was conducted by the Framework 7 EU program called Food4Me. This Food4Me study included a total of 1269 participants in the healthy range of the population from 7 European countries who each completed the internet-delivered intervention over the 6 months of the study [10].

Recently, we conducted an explorative personalized nutrition study that also included the phenotypic flexibility assessment [14]. Here, we could also show a quantifiable health benefit as a result from improved dietary behavior in Dutch seniors receiving personalized nutrition and lifestyle advice as compared to Dutch seniors receiving generic advice. In a group of 59 seniors with sedentary behavior with a BMI between 20 and 30 kg/m^2^ that reported to have good general health, half of the participants received generic advice via a leaflet with the Dutch dietary and physical activity guidelines and the other half of the participants received this same advice but now personalized based on (1) the personal data of the participants with information on muscle health and wellbeing and lifestyle behavior and (2) the personality of the participant. At baseline, both groups received personal feedback on their muscle health and wellbeing. After 9 weeks of intervention, both groups showed quantifiable health improvement. However, the seniors receiving the personalized advice showed additional effects on health, as well as on dietary behavior, such as reduced weight, body fat, waist circumference and resilience, and improved intake of vitamin D and omega-3 fatty acids. Interestingly, it was observed that personalization was especially beneficial for seniors who had a low self-efficacy [14].

It is predicted that in 10 years, the digital evidence-based nutritional or dietary advice will be generated on personal health data including biological (phenotypic flexibility) as well as behavioral measures [42]. This digital advice will be tailored to specific personal preferences and goals, to gain better adherence to one’s optimal diet. The first studies indicate that personalized nutrition indeed results in better adherence to dietary advice, resulting in (short-term) measurable health effects, thus offering opportunities for health promotion. Besides the delivery of next-generation biomarkers that will help in the scientific substantiation of nutrition and food products, phenotypic flexibility is envisioned as a way to provide individual health feedback to persons and as a starting point for personalizing the dietary advice to one’s phenotype, especially for persons in the healthy range of the population.

## Conclusion

Primary prevention is the most effective and affordable means to prevent chronic disease. Emphasizing diet quality and quantity may be the best preventative measure to accomplish long-term personal and societal objectives at every stage of the lifespan. Optimal nutrition, coupled with appropriate physical activity, has been shown to play a central role in decreasing the observable hallmarks of corpulence and obesity. As discussed, the loss of healthspan is apparent by the myriad of non-communicable diseases that are insidious and become manifested by sudden cardiovascular events, liver failure, pulmonary disease, diabetes, and cancers of all types. And even if these severe morbidities are avoided, the loss of cognition, mobility, and social connections are often the greatest fear.

There is consensus among public health workers that nutritional inadequacy and/or overconsumption are influenced by many different factors and reshaping dietary and lifestyle choices will require wider stakeholder collaboration, including but not limited to academic researchers, nutrition product formulators, the medical, dietetic and physical activity professionals, regulators, and public health policy advocates. But the most important investor, and the one against which success could eventually be measured (or not) would ultimately be the individuals comprising in toto the general public. There have been many different public health recommendations provided in the past by the architects of public health and nutrition policies, but results have mostly failed due to lack of engagement with all the stakeholders. The WHO initiative on health promotion will require an integrated approach to the problem.

Lack of commitment by policy- and decision-makers to engage the full breadth of interested parties, a lack of understanding and/or inability to address literacy issues and cultural and communication issues have been major barriers. These factors should be considered and addressed before any health promoting program starts. The success of program(s) will depend on clear goals, expectations and setting milestones to measure or confirm societal health is on an upward beneficial trajectory, maintaining the status quo, or worse, decrements to the overall health. The latter may be characterized by the recent downturn in life expectancies for some developed countries, perhaps due to a lack of commitment by the public to understand the objectives, and to put off until tomorrow any changes in behavior, such as smoking, excess alcohol consumption, sugar/salt/fat intake, lack of exercise, high-stress/high-risk activities, etc. Two hundred years ago, Benjamin Franklin opined “You may delay, but time will not,” [6] and never has that been more true than in the realm of health promotion and healthy ageing.

The nutrition science community must set credible recommendations and communicate those in a way that the public will adopt a health-promoting mindset, promoting eating habits that maximize healthy and enjoyable lives. Nutrition messaging needs to be based on appropriate evidence and communicated in a manner that encourages individuals to adopt healthier dietary and lifestyles, e.g., increased physical activity, energy balance, nutrient density, moderate alcohol consumption, no smoking, and stress reduction, will benefit the individual, the society, as well as the planet.

Understanding what is a “healthy gut”, and the diet and lifestyle changes needed to tweak a person’s microbiome into a beneficial state would have an impact on a person and thus society’s overall pathway to a healthspan that coincides with a lifespan. Progress is being made in “defining a healthy microbiome’ but there is much more to do. It will be important for food and supplement manufacturers and public health experts to avoid mixed messages and to apply the findings as clearly stated opportunities for the person and the populace.

Concepts of personalized nutrition can only reach fruition when the technologies and analytics to measure baseline and perturbations to baseline exist. Resilience and flexibility will need to be known at the individual level, such that subsequent adaptations to insult can be determined and appropriate lifestyle corrections made almost in real time. By tailoring one-on-one instructions to the unique individual, then incrementally, the population should see beneficial results.

Integrated health promotion will need to have the support of the food and nutrition industry. To overcome this challenging societal problem, policy-makers should not only focus on solutions, such as additional taxes or incentives, but also embrace programs that touch many different areas, such as education of school-age children, improved nutrition and labelling literacy, promotion of an active and engaging lifestyle, community and city designs to encourage exercise and access to fresh and wholesome food choices, and a trusted clearing house for nutrition advice and options. However, the most important point will be to encourage individual and societal behavioral change. Eating is both a necessity and a social and cultural phenomena, and adoption will require additional community support from family, friends, health care providers, lifestyle counselors, or social organizations.
